# Supplementary material for: Personal Care and Household Cleaning Product Use among Pregnant Women and New Mothers during the COVID-19 Pandemic
Source: Int J Environ Res Public Health. 2022 May 6;19(9):5645. doi: 10.3390/ijerph19095645 (PMC9104147; doi:10.3390/ijerph19095645)

Supplemental Figure S1. Frequency of use of types of personal care products (a) and household cleaning products (b) during the previous month among women participating in the Maternal Health and Behavior Study (n=320).

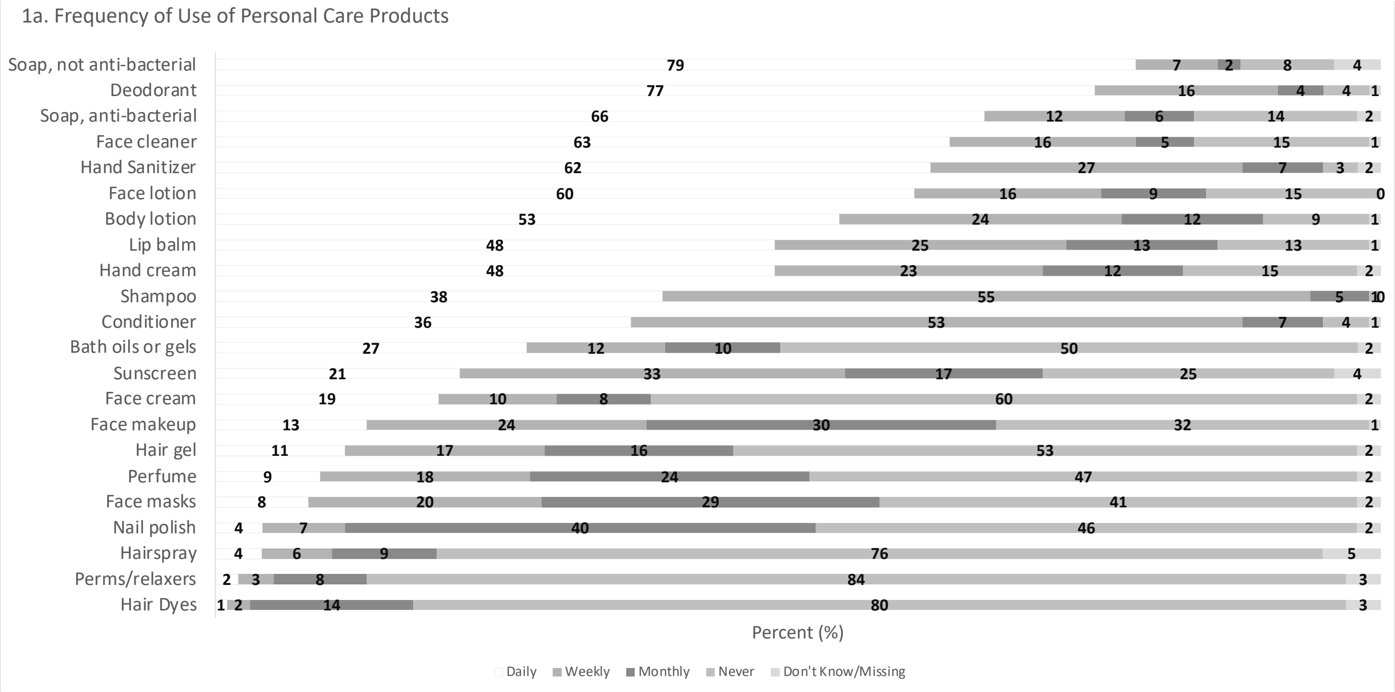

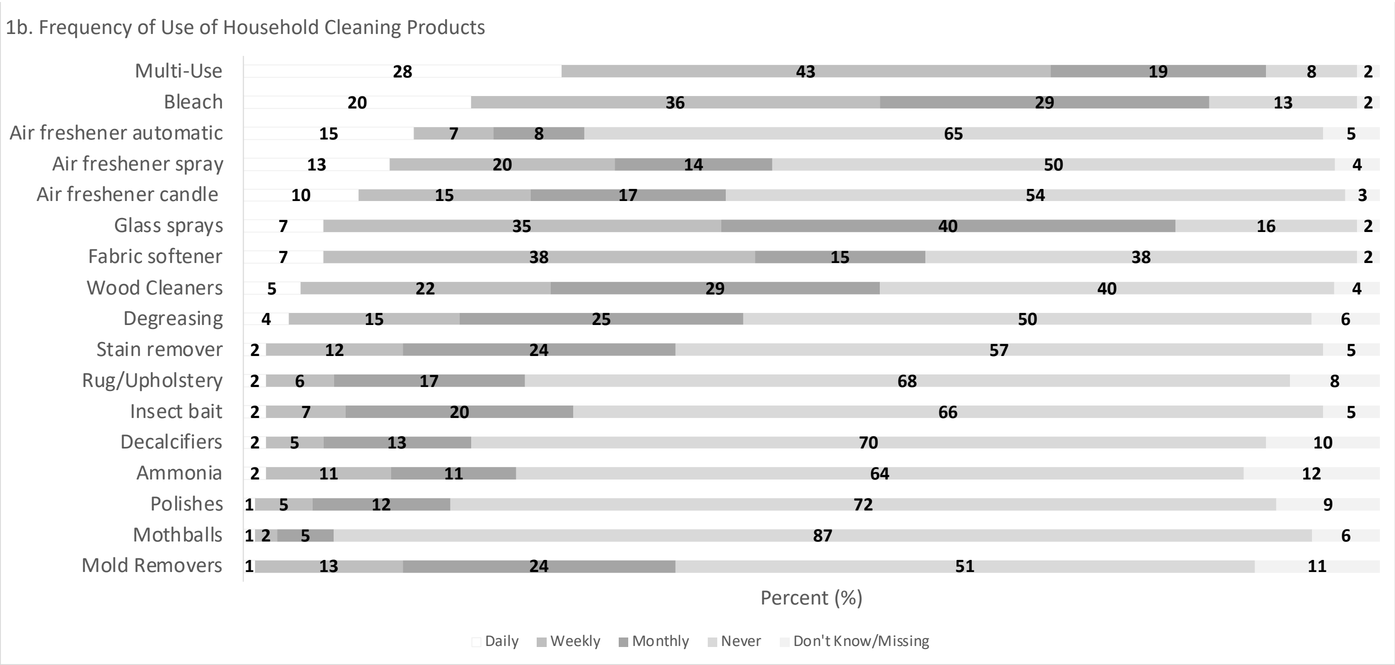

Supplement: Supplementary file 1 [file ijerph-19-05645-s001.zip › ijerph-1683493-supplementary.pdf]
